# The introduction of virtual reality in forensic mental healthcare – an interview study on the first impressions of patients and healthcare providers regarding VR in treatment

**DOI:** 10.3389/fpsyg.2024.1284983

**Published:** 2024-04-30

**Authors:** M. T. E. Kouijzer, H. Kip, S. M. Kelders, Y. H. A. Bouman

**Affiliations:** ^1^Department of Technology, Human and Institutional Behaviour, Centre for eHealth and Wellbeing Research, University of Twente, Enschede, Netherlands; ^2^Department of Research, Transfore, Deventer, Netherlands; ^3^Opentia Research Unit, North-West University, Vanderbijlpark, South Africa

**Keywords:** virtual reality, implementation, forensic mental healthcare, interviews, VR, treatment

## Abstract

**Background:**

Recognizing the potential of Virtual Reality (VR) as a powerful technology to support behavior change, the careful introduction of this technology into treatment settings is essential. This is especially important in vulnerable populations like forensic psychiatric patients. This study aims to gain insight from the impressions of both patients and healthcare providers concerning the integration of VR in practice. The study aims to contribute valuable information that guides the introduction of VR technology, ensuring its optimal use in the complex context of forensic mental healthcare.

**Methods:**

Semi-structured interviews were conducted with healthcare providers (*n* = 10) working at forensic outpatient clinics and forensic psychiatric patients (*n* = 8). All participants experienced VR before the interview. Inductive thematic analysis was employed for analyzing the interview data.

**Results:**

Patients valued the unique opportunity to simulate personal experiences in VR scenarios and reflect on them with healthcare providers. In addition to positive first impressions, areas for improvement were identified, including the wish for enhanced realism and reduced physical discomfort while immersed in VR. Finally, important factors contributing to the successful introduction of VR were identified. For example, taking into account psychological distress experienced by patients or supporting healthcare providers with implementation resources.

**Conclusion:**

The integration of VR into forensic mental healthcare holds great potential for behavior change. However, its immersive characteristics also increase the chance of amplifying psychological distress. This emphasizes the need for caution when using VR– especially when a vulnerable patient group is subjected to triggering scenarios. This study advocates for a gradual introduction of the technology and provides valuable insights into essential elements for this introduction in clinical practice. It highlights that even the initial step of integrating VR into practice – the introduction phase – demands careful planning and a personalized approach. This underscores the need for ongoing refinement and a systematic approach to the overall implementation of VR. These efforts are crucial to fully realize its potential in clinical practice.

## 1 Introduction

Forensic psychiatry, a specialized field within mental healthcare, is focused on the assessment and treatment of individuals whose behavior has led or could lead to offending, often complicated by one or more psychiatric disorders (Mullen, 2000). For forensic psychiatric patients, a notable difficulty with recognizing and regulating their emotions is often evident, presenting a key area for intervention and exploration (García-Sancho et al., 2014; Roberton et al., 2015; Garofalo et al., 2018). This opens up possibilities for innovative approaches to enhance emotion awareness and regulation. Recent studies have highlighted Virtual Reality (VR) as a potentially suitable treatment tool for addressing emotion regulation challenges (Kip et al., 2019a,b; Tuente et al., 2020; Smeijers et al., 2021). VR offers the possibility to immerse patients in a unique virtual environment that can simulate real-world scenarios through a head-mounted display and 3D graphics (Skip et al., 2018). Particularly, interactive VR shows promise in bridging the gap between the treatment room and the outside world (Botella et al., 2017; Tuente et al., 2020). In interactive VR, patients are immersed in real-world scenarios that allow them to experience a sense of presence while interacting with the virtual world as if they were physically present within it (Botella et al., 2017; Tuente et al., 2020). VR allows patients to experience a sense of belonging within a virtual body but also actively contributes to regulating emotional well-being (Slater et al., 2008, 2010; Ventura et al., 2018; So et al., 2022). It offers patients the unique opportunity to engage in therapeutic activities, providing them with a safe space to practice new behaviors and coping strategies (Botella et al., 2017; Sygel and Wallinius, 2021).

Interactive VR offers various treatment opportunities within forensic psychiatry. It can be applied to expose patients to stimuli or situations that can elicit an emotional response such as fear or anger (Botella et al., 2017). By gradually exposing patients to these scenarios in a safe and controlled environment, they can learn to better manage their anxiety, fear, or aggression by practicing coping strategies (Botella et al., 2017; Baniasadi et al., 2020; González Moraga et al., 2022). For example, a patient with emotion regulation issues could be exposed in a role-play to a relatively strict police officer and practice their relaxation and communication skills. Besides exposure, VR can be used as a tool for assessment of individuals’ risk of violence or re-offending, e.g., by recreating virtual scenarios that may trigger problem behavior that resembles behavior outside of the treatment room, allowing healthcare providers to observe and evaluate patients’ reactions and potential risk factors in real-world situations (Renaud et al., 2014). Despite the growing awareness of the possibilities offered by VR, the use of VR in practice remains in its infancy (Garrett et al., 2018; Smith et al., 2020).

While research has demonstrated the potential benefits of VR within forensic psychiatric patients, the practical implementation of VR in practice often lags behind (Kouijzer et al., 2023). This process of implementation, crucial for the effective use of VR, encounters obstacles because of implementation barriers like limited familiarity with the technology, resistance to change, and technological apprehension (Kouijzer et al., 2023). To conquer these challenges, a thorough introduction to VR is advised (Kouijzer et al., 2023). From an implementation perspective, introducing the new technology among the people who need to work with it, such as healthcare providers and patients. The introduction of VR refers to familiarizing healthcare providers with the technology before its actual application in treatment and letting patients gradually acclimate to VR during the initial treatment sessions (Kouijzer et al., 2023). From an ethical point of view, a careful introduction of new technology is important, especially in this unique and vulnerable target group of forensic mental healthcare patients where transgressive behavior and a variety of psychiatric disorders play an important role (Fassaert et al., 2016). The introduction of VR technology itself requires cautious consideration due to its immersive and intrusive characteristics (Baniasadi et al., 2020). Because of these characteristics, VR can elicit intense emotional and psychological responses (Fromberger et al., 2014; González Moraga et al., 2022). The virtual environments and scenarios created in VR can be highly realistic, exposing patients to situations that could trigger their problem behavior or simulate traumatic experiences. Recalling traumatic experiences or memories can be highly effective in treatment, as demonstrated in EMDR therapy (Portigliatti Pomeri et al., 2021). However, it may also lead to heightened psychological distress, which could have unintended consequences on the mental well-being and safety of patients and healthcare providers involved in the VR treatment (González Moraga et al., 2022). This indicates the importance of a balanced approach that weighs potential therapeutic gains against the potential risks and fosters a careful introduction into practice (González Moraga et al., 2022).

To determine how to introduce VR in practice, it is important to consider the perspective of stakeholders in the development and implementation phase of the technology (van Gemert-Pijnen et al., 2018). Ventura et al. (2018) underline the importance of a focus on user experience in this introduction, especially when introducing a new technology as a treatment tool. They emphasized that future studies should focus on the psychological aspects and personal feelings of participants during a full-body immersion in VR (Ventura et al., 2018). By understanding the first impressions of both healthcare providers and patients regarding the VR intervention, their initial reactions and perceptions are explored. These play an important role in shaping the overall implementation strategy. They offer insight into how stakeholders perceive the technology’s potential benefits and challenges, allowing for a more informed and effective integration process and thus increasing the chances of adoption and long-term use (van Gemert-Pijnen et al., 2018). Additionally, involving end-users prevents a top-down approach in which researchers or software developers dictate how VR should be introduced. It allows for optimal fit between the needs and wishes of patients and healthcare providers and the technology, making sure that VR is of added value for them (Kip et al., 2019a,b).

### 1.1 The current study

Given the immersive characteristics of VR technology, the current study places a significant focus on understanding the perspectives of end-users to navigate the careful introduction of this technology in a vulnerable forensic population. The primary objective is to gain insight into patients’ and healthcare providers’ initial impressions and perspectives regarding the use of VR in forensic mental healthcare. By prioritizing the examination of user experience, the study aims to contribute to a balanced and informed approach that weighs therapeutic gains against potential risks for patients and healthcare providers, fostering a careful integration of VR technology in the treatment of forensic psychiatric patients. To achieve this overall aim, the following research questions will be addressed:

1a. What are the initial impressions of patients regarding their immersive experience within the VR intervention?

1b. What are the initial impressions of healthcare providers regarding the dashboard and possibilities of the VR intervention?

2. To what degree do patients report changes in their psychological distress levels during VR immersion?

3a. What critical factors should be considered when introducing VR in treatment, according to patients?

3b. What critical factors should be considered when introducing VR in treatment, according to healthcare providers?

## 2 Materials and methods

### 2.1 Study setting

This study focused on investigating the first impressions of patients and healthcare providers regarding a virtual reality intervention in two Dutch forensic mental healthcare organizations: Transfore and De Waag. Both organizations provide treatment for aggression regulation and sexually transgressive behavior to forensic psychiatric patients who either committed or are at risk of committing a criminal offense due to psychiatric problems. Transfore has multiple treatment locations in the east of The Netherlands and offers treatment to over 1,500 in-and-out patients every year. De Waag is an outpatient clinic with 12 treatment sites throughout The Netherlands. They offer treatment to around 7,000 patients a year. Ethical approval was obtained from the Ethics Committee of the University of Twente (Behavioral, Management, and Social Sciences, number 210645). This qualitative study adheres to the consolidated criteria for reporting qualitative research (COREQ) (Tong et al., 2007).

### 2.2 The intervention

**Figure 1:**
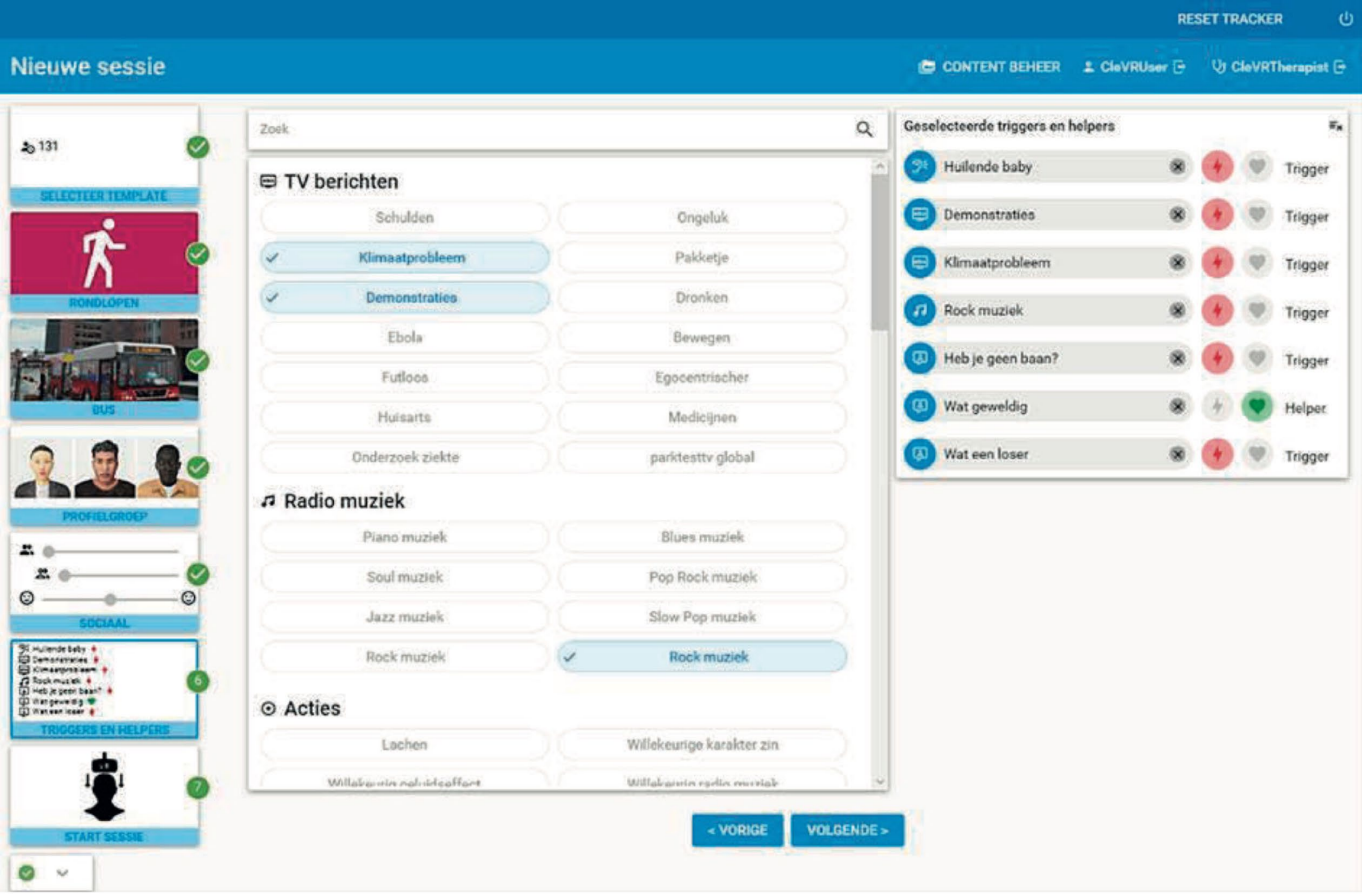
Dashboard of the VR intervention “Triggers & Helpers”.

**Figure 2:**
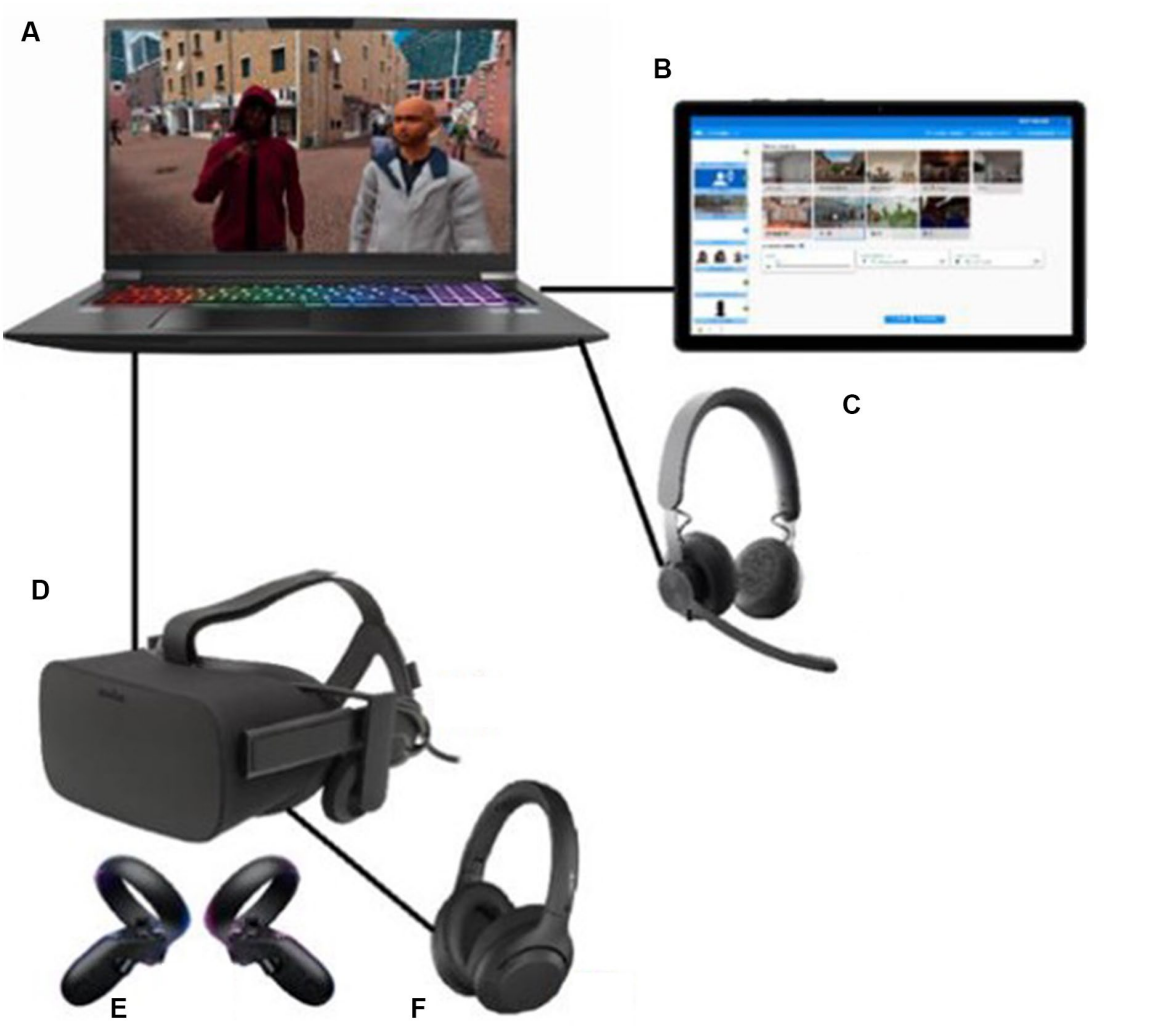
Setup of VR consisted of laptop **(A)**, tablet with dashboard of “Triggers & Helpers” application **(B)**, voice-morphing microphone **(C)**, VR head-mounted display **(D)**, VR controllers **(E)**, noise-canceling headphones **(F)**.

**Figure 3:**
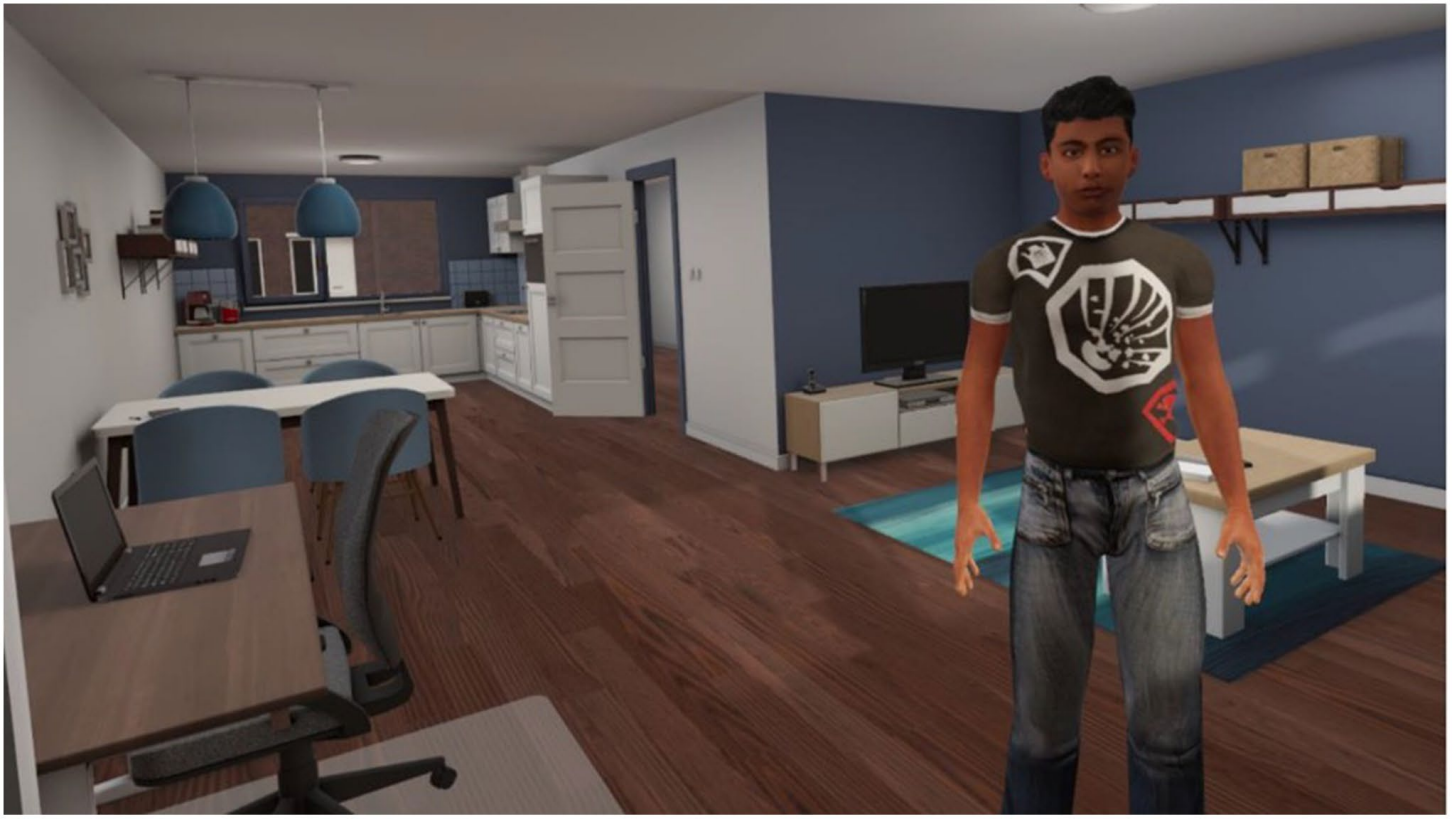
Screenshot of a virtual living room in which the patient can perform a role-play with a virtual character (^©^CleVR).

**Figure 4:**
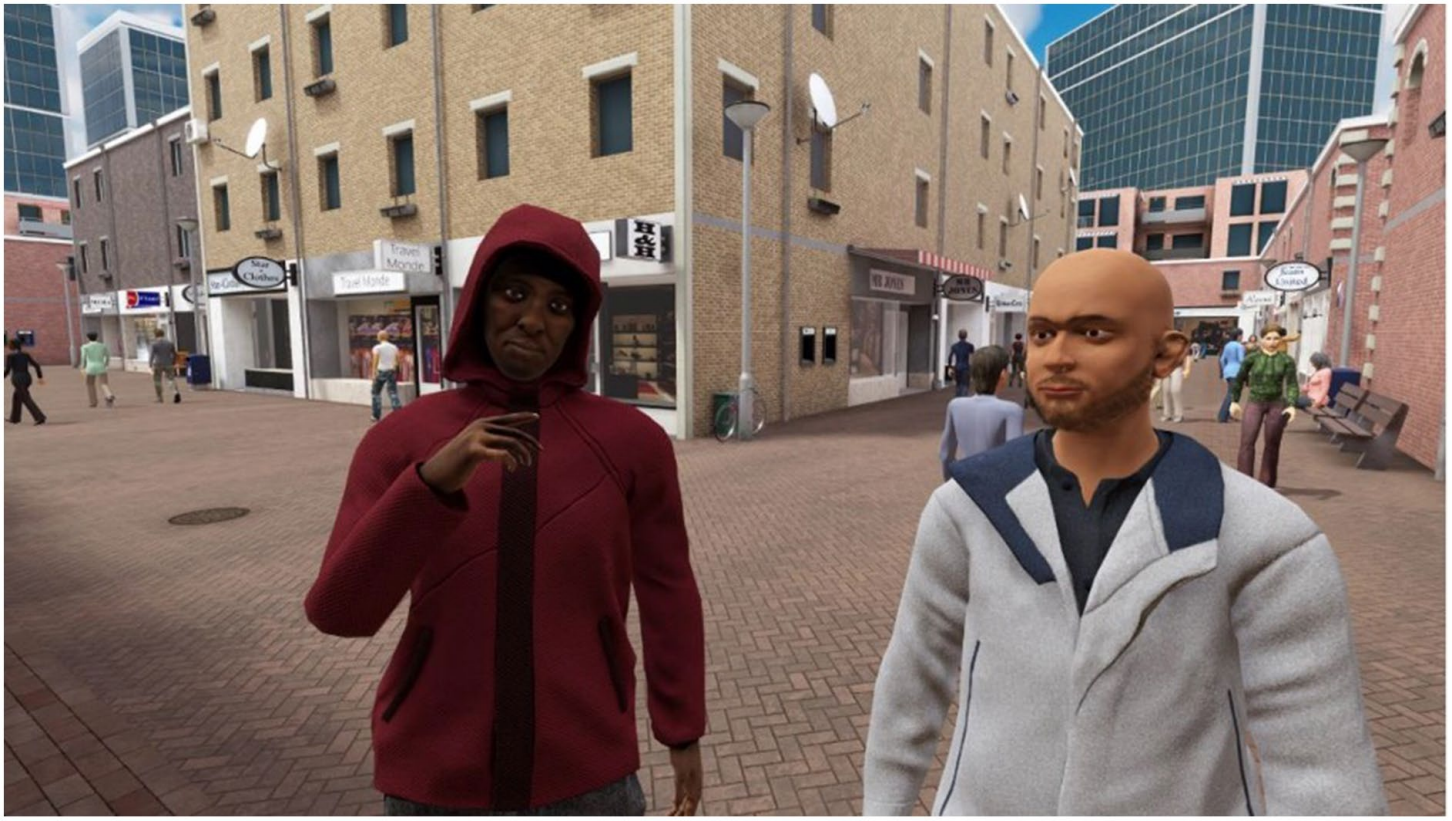
Screenshot of a virtual shopping street in which the patient can walk around using a controller or can perform a role-play with multiple virtual characters (^©^CleVR).

The interactive VR intervention that was applied in the current study is called ‘Triggers & Helpers’. The VR software was developed by CleVR. The patient wears a head-mounted display and noise-canceling headphones. While being immersed in a VR scenario, the patient can walk through a broad range of virtual environments such as a supermarket, a shopping street, or a home environment, using a controller. Additionally, the patient can conduct a role-play with virtual characters. This character is portrayed by the healthcare provider using a voice-morphing microphone. The healthcare provider can assume a broad range of virtual characters with different types of voices, allowing for a highly personalized experience. They can control the movements, facial expressions, and body language of the character using a dashboard (see Figure 1). Here, they can also enable changes in environments, such as increasing the number of passers-by on a street or characters that enter a virtual room during the scenario. In Figure 2, the setup of the VR technology is displayed. With this technology, a personalized VR scenario can be developed for different types of patient needs. In Figures 3, 4, screenshots of two virtual environments of the application “Triggers & Helpers” by CleVR are provided.

### 2.3 Participants

Interviews were conducted with patients and healthcare providers. Inclusion criteria for patients were: they had (1) to be fully informed about the study and willing to participate voluntarily, (2) no prior experience with the VR intervention “Triggers & Helpers” to elicit their first impressions, (3) followed some form of aggression regulation treatment in an out-patient setting, and (4) approval from their healthcare provider, who indicated that the immersion in VR would not be uncomfortable or damaging for the patient or their treatment goals. For safety reasons, their healthcare provider was present during the VR experience and interview. For healthcare providers, inclusion criteria were that they were (1) currently working in forensic mental healthcare, (2) involved in any type of aggression regulation treatment for forensic outpatients, and (3) potential end-users of the VR intervention “Triggers & Helpers”. Patients and healthcare providers who fail to meet the inclusion criteria will be excluded from the study. For the patient group, (1) individuals with epilepsy, (2) severe dizziness, or (3) severe visual impairments, as well as those experiencing (4) active psychosis or (5) another form of crisis as assessed by their healthcare provider, will be excluded from the study. These measures have been implemented to ensure the safety and well-being of participants during the VR immersion.

The recruitment of participants was carried out by convenience sampling, a nonrandom sampling method where members of the target population meet certain practical criteria, such as easy accessibility to the researcher, availability at a given time, or willingness to participate (Dornyei, 2007; Given and Lisa, 2008). Suitable healthcare providers and patients were identified by the project team. This team consisted of two researchers, three healthcare providers, two former patients, and one policy officer working on the development and implementation of the VR intervention. The team identified a list of 10 potential healthcare providers working in forensic mental healthcare organizations that fit the inclusion criteria. The team paid attention to including a broad range of participants, working in different organizations, and working with different aggression regulation treatment groups to ensure that a variety of healthcare provider perspectives were included. These healthcare providers were approached by email by the researcher (MK) with the request to participate in an interview and all agreed. Next, these healthcare providers were asked to select suitable patients out of their caseload, inform them about the study face-to-face, and ask if they wanted to participate. Patients were intentionally selected from two distinct forensic mental healthcare organizations, representing a range of age groups, and participating in different types of aggression regulation treatment: individuals from a ‘regular’ aggression regulation group and those from a group tailored for individuals with mild intellectual disabilities. This deliberate inclusion of participants from diverse backgrounds and varying intellectual abilities ensured a comprehensive representation of potential end-users of the VR application. In total, 8 patients were approached by their healthcare provider, and all agreed to participate in an interview. No approached patients or healthcare providers declined participation or dropped out during the study. A total of 18 interviews were conducted by one researcher (MK) between March and July of 2021. Participant characteristics are presented in Table 1.

**Table 1:** Participant characteristics.

Characteristics	Patients – *N* (%)	Healthcare providers – *N* (%)
Gender		
Male	8 (100)	2 (20)
Female	0 (0)	8 (80)
Age		
20–29 y	1 (12,5)	
30–39 y	4 (50)	
40–49 y	2 (25)	
> 50 y	1 (12,5)	
Treatment type		
Aggression Regulation group	6 (75)	
Aggression Regulation group for mild intellectual disabilities	2 (25)	
Function		
GZ psychologist		6 (60)
Forensic nurse		2 (20)
Occupational therapist		1 (10)
Forensic remedial educationalist		1 (10)
Prior experience with VR		
Used VR in treatment	0 (0)	8 (80)
Never used VR in treatment	8 (100)	2 (20)

### 2.4 Data collection

Data were gathered through in-depth, semi-structured interviews by one researcher (MK). Semi-structured interviews refer to a qualitative research method that is conducted with one respondent at a time, employing open-ended questions, often accompanied by follow-up ‘why’ and ‘how’ probing questions. This dialogue allows to delve into unforeseen and important issues for the research topic (Adams, 2015). Semi-structured interviews are often used in exploratory research when not much is known about a specific topic and are especially suited when multiple interview questions require follow-up queries in the form of probing questions that ask about the independent thoughts of each participant (Adams, 2015).

Before the interviews took place, patients and healthcare providers were informed by the researcher (MK) about the goal of the interview, the reasons for and interests in the research topic, and signed the informed consent form. In addition, patients were immersed for the first time in different virtual environments and had the opportunity to explore these environments by walking through them or talking to virtual characters in a role-play setting. While patients were immersed in the VR intervention itself, healthcare providers were informed by the researcher about the possibilities of VR and viewed screenshots of all available virtual environments, characters, and the dashboard before the interview took place.

The interview schedule for patients consisted of 7 open-ended questions with accompanying sub- and probing questions. A pilot interview was conducted by one researcher (MK) to refine the schedule and improve questions whenever necessary. The interview questions centered around patients’ first impressions of VR and any suggestions for improvement: *“What did you think of the VR experience?”.* Specific attention was focused on patients’ experience of psychological distress during the VR scenarios, this was asked during the interviews; *“On a scale of 1 to 10, with 1 being completely relaxed and 10 experiencing extreme stress, how high was your level of distress?”*. During the interview, patients were also queried about their perceptions of using VR in treatment and any important considerations for its introduction into practice. Sample questions include: “*To what extent would you like to use this VR application in treatment?”* and *“How would you apply this VR application in treatment?”.* Additional probing questions were asked to elicit an extra level of detail via verbal prompts to clarify, elaborate, or explain a prior answer to an interview question that the participant had already given. The patient interviews, after the VR immersion, took place face-to-face at the forensic hospital and lasted an average of 23 min.

The interview schedule for healthcare providers consisted of 9 open-ended questions with accompanying sub- and probing questions. An additional pilot interview was conducted. The interview questions focused on the first impressions of healthcare providers of the VR dashboard and opportunities in treatment. Additionally, any points for improvement for the VR system were discussed. A sample question: *“What is your initial impression of using the VR dashboard?”*. Furthermore, healthcare providers were asked about their thoughts on the use of VR in treatment and any points of attention for the introduction of VR in their work practice, for example: “*How would you apply this VR application in treatment?*”, and *“What would that look like in practice? Can you give some examples?”*. Additional probing questions were asked in the healthcare provider interviews as well. The interviews with healthcare providers took place via Zoom, an online meeting program, due to the worldwide Covid pandemic that limited treatment on site. These interviews lasted an average of 47 min. Both types of interviews were recorded with a voice recorder. The interview schedules for patients and healthcare providers are provided in Tables A1, A2.

### 2.5 Data analysis

The audio recordings of the semi-structured interviews were transcribed verbatim and anonymized by one researcher (MK). To this qualitative data, thematic analysis was employed. This analysis provides an accessible and systematic procedure for generating codes and themes from the qualitative data (Clarke and Braun, 2017). A coding scheme was iteratively created by one researcher (MK), while another researcher (HK) remained consistently engaged in the process, providing continuous oversight. First, the transcripts were read carefully to familiarize with the data and identify meaningful fragments related to one of the research questions. These fragments were linked to codes that captured interesting features of the data, relevant to the research questions. These different codes were building blocks for themes, a larger pattern of meaning. These themes and related codes were summarized in a coding scheme. The codes were selected by the method of constant comparison between interview fragments (Glaser, 1965; Boeije, 2002). The coding scheme was adapted throughout this process. This first version of the coding scheme was thoroughly discussed with another researcher (HK) and an improved version was used to code the other transcripts. The transcripts were coded and compared until the saturation point, at which no new codes relevant to the research questions were identified in the data (Glaser, 1965; Boeije, 2002). One researcher (MK) coded the fragments and discussed them with another researcher (HK) in case of any doubt. Definitions of codes were adapted throughout the process.

## 3 Results

### 3.1 First impressions of VR according to patients

To answer the first research question, the initial impressions of patients regarding their immersive experience within the VR intervention are explored. This category refers to the judgments or perceptions of patients while being immersed in VR for the first time. The related codes mentioned by patients are reported and defined in Table 2.

**Table 2:** First impressions of VR according to patients.

Code	Definition	N*int*^a^	N*tot*^b^
Positive first impressions			
Feelings of enjoyment	Situations or interactions that bring emotions of joy, satisfaction, curiosity, or excitement to patients while using VR.	8	18
Sense of presence	Subjective experiences of the extent to which patients were fully engaged and immersed in a virtual environment.	7	16
Points of improvement			
Lack of sense of presence	Lack of subjective experiences of the extent to which patients we not fully engaged and immersed in a virtual environment.	7	10
Unnatural movement	Aspects that could be improved related to navigating within a virtual environment, allowing users to explore and interact with this environment.	7	9
Feelings of discomfort	Unpleasant sensations experienced by patients while being immersed in VR, such as dizziness or increased body temperature.	8	8

a The number of interviews the quote was mentioned in (N*int*).

b The number of times the code was mentioned in all interviews with patients (N*tot*).

#### 3.1.1 Positive first impressions

All patients mentioned positive first impressions regarding VR. Their first experience was regarded as fun, increasing curiosity, creating possibilities, and overall experiencing *feelings of enjoyment*. A few patients described the VR intervention as a great, innovative technology that creates possibilities to practice new behavior in real-life situations that can be re-created in a virtual world. Patients mentioned that beforehand, they did not know what to expect from the intervention. However, they were excited and curious to try it out; *“I did not know what to expect. I never experienced it before, but I was curious, and it was awesome. (…) It’s good to practice in VR how to find peace again. I would highly recommend this.”* (P8)

Most patients were positively surprised by how easy it was to recreate situations with the healthcare provider and experience them in VR, in contrast to only talking about an experience during treatment. To illustrate; “*It’s great that you can make things clearer with this. Explaining experiences [face-to-face] is more difficult than showing it [in VR].”* (P5)

Regarding the *sense of presence*, the feeling of being fully engaged and immersed in the virtual environment, most patients described that they felt as if they were walking through the virtual environment and talking to a virtual character. Some patients described feeling present as if being present in a game; *“Physically you are in the [treatment] room, but mentally you are in VR. It’s a strange feeling. It really can be compared to a game”* (P3). Additionally, the vast majority of patients described heightened alertness and an increased sense of situational awareness. Patients shared that the unfamiliar environment prompted them to prepare themselves to act upon unexpected situations. For instance, there was a sense of curiosity among patients about the potential outcomes when they encountered virtual characters that initiated conversations. One patient illustrated:

*“I have to be alert to all the people walking by. For example, when suddenly a man comes very close to me and unexpectedly takes his phone out of his pocket, then I notice for myself that I become very alert to these small movements. That's something from my past.”* (P8)

A factor that influenced the feeling of presence was the level of experienced realism. Aspects that contributed to this feeling were details in the environment; *“I thought the environment was portrayed quite realistically. I once saw a garbage can or an air conditioner hanging in the corner at the shopping street. Those are details I pay attention to.”* (P1). In addition, according to some patients, the feeling of realism increased when the virtual characters started an interaction with the patient. Patients had the feeling that they had to react, had to shake their hand, or had to step aside when a character approached. As explained by some patients; They know that it is a simulated environment, however, when immersed in VR, they are forced to act upon the virtual situation; *“It feels different. You know it’s fake, but it looks realistic. Your brain will believe that you are in VR. It looks real.”* (P5)

#### 3.1.2 Points of improvement

Patients provided both, positive and negative first impressions of VR during the study. While some patients had predominantly positive experiences regarding their sense of presence, others highlighted areas for improvement in this matter.

A frequently mentioned concern among patients centered on the *lack of a sense of presence* during instances where they focused on the details in the virtual environment. For instance, the sense of presence decreased when patients directed their attention toward virtual characters. One patient expressed, “*I do not experience it as real; it feels unrealistic. There is still very little feeling or emotion [while conversing with a virtual character]. So, I cannot see if he means what he says. It is now very fake and superficial.”* (P7). Another aspect of VR that decreased the sense of presence was related to the appearance and movement of virtual characters. Some patients found the virtual characters to be lacking in emotional expression, describing their body language, appearance, and physical movements as robotic or unnatural. One patient elaborated, *“Now the person facing you is still robotic. It must seem somewhat real to use for people with aggression problems. The movements and appearance feel very unnatural.”* (P2). This unrealistic portrayal of characters made it challenging for patients to fully engage in interactions with them. Despite this, some patients acknowledged that these VR characters could still be used to simulate realistic situations since they felt like they needed to respond in a conversation when a character talked to them. Finally, patients expressed a desire for more realistic details in both the characters and environment to increase the sense of presence. One patient wished for a higher level of fidelity and accuracy in the visual and auditory aspects of the virtual environment. They suggested, *“It would be nice if you could also look in the store through the window or hear some more background noises. Maybe you can walk everywhere and hear sounds from the houses above or birds flying and chirping above you.”* (P6)

An additional negative first impression was related to the *unnatural movement* in VR that was experienced by patients while walking through an environment. They experienced the slow walking pace as irritating and unnatural. The slow movements did not match the physical movement patients would be able to execute in real life. They described this as hindering the feeling of being completely immersed in the virtual world.

*“It’s different. You stand still yourself [in real-life], but it feels like I’m walking [in VR]. Everything I see has to be processed by my brain. So basically, my brain is being fooled and that’s why it feels so weird in my body. The reality does not match and that makes me dizzy for a while.”* (P7)

The unnatural movement experienced by patients increased the *feelings of discomfort* for four patients in VR. They mentioned that they experienced dizziness during VR because of it. As one patient illustrated:

*“It’s different. You stand still yourself [in real-life], but it feels like I’m walking [in VR]. Everything I see has to be processed by my brain. So basically, my brain is being fooled and that’s why it feels so weird in my body. The reality doesn’t match and that makes me dizzy for a while.”* (P7)

Additional aspects that increased discomfort were increased body temperature during the VR session. This was mentioned by one patient who explained that this was because of the feeling of being “enclosed” by the head-mounted display and the noise-canceling headphone.

Most patients agreed on these points of improvement, however, two patients did not have any specific suggestions for improvement for the current version of the VR intervention.

### 3.2 First impressions of VR according to healthcare providers

To answer the first research question regarding the first impressions of healthcare providers, their initial judgments and perceptions regarding the current version of the VR dashboard were explored. The codes mentioned by healthcare providers are reported and defined in Table 3.

**Table 3:** First impressions of VR according to healthcare providers.

Code	Definition	N*int*^a^	N*tot*^b^
Personalization	The customization and adaptation of VR scenarios to suit the preferences, needs, and characteristics of individual patients.	5	7
User-friendly	The ease of use, accessibility, and intuitive nature of the VR software.	3	3
Preview of VR scenario	A brief demonstration or glimpse that is provided to the users that shows the VR environment and virtual characters.	2	3
Variety in environments	The presence of diverse and suitable settings in which a scenario can take place within the VR software.	1	1
Wish for more realistic detail	The desire to have a higher level of fidelity and accuracy in the visual and auditory aspects of the virtual environment.	1	1

a The number of interviews the quote was mentioned in (N*int*).

b The number of times the code was mentioned in all interviews with healthcare providers (N*tot*).

#### 3.2.1 Positive first impressions

Overall, healthcare providers positively evaluated the great variety of options to create and *personalize* a VR scenario, such as the extended list of VR characters and environments. The software was reviewed as *user-friendly*, providing the possibility to create a VR scenario with a structured, step-by-step approach. As one healthcare provider illustrated; *“I find it useful that you can see on the left [of the dashboard] at which step you are and what the next step is. All the expansions are nice. This allows more variations to be made in the VR characters. That is nice for patients to be able to personalize it. I find it user-friendly. It’s easy and even for me it’s doable [to set up a VR scenario].”* (H8). Healthcare providers appreciated the option to go back and forward between the steps to adjust the VR scenario to fit with the treatment goal. To illustrate:

*“My first impression is that it is user-friendly. It is useful and nice that it [setting up a VR scenario] goes step by step. I am surprised about all the options you can choose from. It’s super comprehensive. I don’t miss anything in terms of environments.”* (H9)

#### 3.2.2 Points of improvement

In addition to positive first impressions, healthcare providers mentioned some points of improvement for the VR intervention. Some mentioned that it would be valuable to have the option to see a *preview of the VR scenario* before actually immersing patients in the VR environment. They mentioned that it can be difficult to imagine what the scenario would look like for the patient when being submersed in VR;

*“It would be nice if you as a healthcare provider could see a concept of the VR scenario, perhaps by clicking on a special button at the last step, to see what that session will really look like in VR. Then you can see, for example, that the police officer is placed over there and the cashier is really behind the cash register. That you can see a preview of the session that you created before you click ‘play’. Then you can easily adjust if something isn’t right yet.”* (H6)

Regarding the VR environments, most healthcare providers appreciated the variety of options. However, a healthcare provider did mention that it would be great if there would be more *variety in environments*, besides an office space, since most patients work in more physical workplaces, such as construction sites. A variety in characters was already achieved and appreciated. However, healthcare providers mentioned that a filter to this extensive list of VR characters would make it easier to personalize a VR scenario. For example, find a fitting character for a role play, such as a police officer or an older gentleman. Adding a filter for the characters’ profession, age, gender, or length was mentioned.

Additionally, the virtual living room and kitchen were regarded as too clean and neat. According to one healthcare provider, this would not match the realistic living situation of most patients. They would prefer *more realistic details*, such as a living room that is messier and less clean. As someone illustrated; *“What I did notice is that the VR environments look too neat. It should be a bit messier to be realistic.”* (H10)

### 3.3 Subjective psychological distress

To answer the second research question, the experienced psychological distress of patients during a VR session is explored. While patients were immersed in different VR scenarios, they were asked to rate their subjective psychological distress on a scale from 1 to 10, with 1 being completely relaxed and 10 experiencing extreme stress. For an overview of their experienced subjective psychological distress during the VR session, see Table 4.

**Table 4:** Subjective psychological distress on a scale from 1 to 10^a^.

Patient nr.	Task 1	Task 2	Task 3	Task 4
1	2	2	2	3
2	1	1	2	2
3	2	2	2	2
4	1	1	1	1
5	6	5	5	6
6	6	7	8	8
7	1	2	2	4
8	2	1	6	6

a A score of 1 indicates that the patient was being completely relaxed and a score of 10 indicates extreme stress experienced by the patient.

In general, patients mentioned that their experienced distress was relatively low during the start of the VR session. Six out of eight patients mentioned that their distress level was 2 out of 10 or even lower at the start of the VR session. The other two patients rated their distress level with a 6 as a starting point. Most patients mentioned that this psychological distress did not increase further during the VR session. However, two patients reported a notable increase in distress during interactions with the virtual environment. These patients mentioned an increase from level 1 to level 4 or 6 during this interaction. For example, patients mentioned an increase in distress when a virtual character stood in front of them and started moving or talking. They explained this increase by having the feeling that they had to be more alert and were forced to react and deal with the situation. As illustrated by a patient; *“The stress is a bit higher because someone [a virtual character] is facing me. I know it is not real, but I still have the feeling that I have to deal with him.”* (P7)

Additionally, patients did not know what to expect during the VR session. This experience of uncertainty increased distress and forced patients to be extremely attentive to the situation and their behavior. One patient illustrated the consequence of this increase:

*“It forces me to react differently than I normally would. Normally I would run away, but now I have to stay and keep calm. I have to go along with a situation that is unfamiliar to me, that I actually don't feel comfortable with at the time. Someone who suddenly stands so close to me and makes unexpected movements. Those kinds of moments actually only occur in a bad dream, when you completely freeze. That's what actually happens.”* (P8)

### 3.4 Possibilities of VR in treatment according to patients

To answer the third research question on critical factors to consider when introducing VR in treatment, the opinions and preferences of patients on if and how VR could be applied in treatment are provided. The viewpoint of patients regarding their willingness to use VR and the potential applications of VR play an important role in the introduction of VR in practice. By exploring their opinion, it becomes possible to identify key areas where VR can be most effectively applied, as a first step in the integration into treatment. The codes mentioned by patients are reported and defined in Table 5.

**Table 5:** Possibilities of VR in treatment according to patients.

Code	Definition	N*int*^a^	N*tot*^b^
Willingness to use VR	Indication of the extent to which patients are ready and willing to use VR in their own treatment.	5	7
How to use VR in treatment			
Possibility of reflection	The capacity to analyze and evaluate one’s behavior and responses in VR scenarios that recreate situations experienced by patients in the real world.	8	10
Triggering fear	The intentional use of VR scenarios to elicit fear or a sense of anxiety in individuals by exposing them to simulated situations or stimuli that evoke a fearful response.	3	3
Triggering aggression	The intentional use of VR scenarios to elicit aggressive thoughts, emotions, or behaviors in individuals by exposing them to simulated situations or stimulate that evoke aggression.	3	3

a The number of interviews the quote was mentioned in (N*int*).

b The number of times the code was mentioned in all interviews with patients (N*tot*).

#### 3.4.1 Willingness to use VR in treatment

The opinions of patients regarding their willingness to use VR in their treatment differed. Five out of eight patients were willing to use VR in their own treatment. They were excited to use new, innovative technology. Some patients mentioned that they would also recommend this technology as a treatment tool to other patients; *“Yes, I would definitely like to use this. I would really recommend it to others as well. (…) You can actually see the effects immediately. Thus, treatment-oriented, I think VR could be more effective [than traditional treatment]”* (Skip et al., 2018).

However, three out of eight patients mentioned that they would not prefer to use VR. They also mentioned that this form of technology would not be suitable for all patients. Firstly, a patient mentioned not being willing to use VR because he felt a lack of technical skills and experience with innovative technology created a barrier. He expressed a clear preference for practicing behavior in real life; *“I prefer to have a real person in front of me. I cannot imagine talking to a virtual person. It just does not feel real. I know this is fake. I find digital and virtual communication harder to understand.”* (P7). Another patient agreed, he described difficulty in acting in VR as if it were a real situation, feeling ‘insensitive’ to the virtual scenarios. However, the patient expressed that others might feel more receptive toward VR.

#### 3.4.2 Treatment possibilities with VR

While three out of eight patients did not prefer to use VR in their treatment, all patients mentioned several opportunities on how VR could be applied in treatment. Patients had a clear idea of the possibility of VR to recreate real-life experiences and reflect on these experiences together with a healthcare provider. They explained that VR provides the possibility for healthcare providers to see what they normally cannot, creating an important *possibility of reflection* in real-time. One patient illustrated:

*“It’s nice that it’s possible to recreate my experiences or what I’m going through in daily life [in VR] and then reflect on that. If you simulate my experiences in the [VR] system, the healthcare providers can see what they normally don’t see. You can tell that you fought with someone yesterday, and tell them exactly what happened, but then it is still a guess for that person how it really went. [In VR] you can simulate that situation together and reflect on it.”* (P7)

Patients mentioned two concrete examples using VR as a tool for exposure in treatment. Firstly, VR can be implemented in the treatment of patients with agoraphobia, patients who feel insecure when interacting with others, or any other kind of *fear that patients can be exposed to* in VR. They expressed that a triggering or fear-inducing situation will be easier to experience step-by-step in the safe environment of the virtual world because VR allows patients to remove themselves from the virtual scenario whenever their distress rises too high. They can be willing to practice this in the controlled environment of VR, however, they might be too afraid to practice this in real life. One patient illustrated;

*“You could use it for role play. For example, if someone is afraid of something, you can expose them to it in a controlled way. I do think that could be useful. You can make someone take a step forward that they might be too afraid of or too shy to do in real-life.”* (P6)

Second, according to patients, VR can be used for patients who display aggressive behavior toward other people. Patients might be *exposed to situations that trigger their aggression* and, they can react to this in a safe, controlled, virtual environment. As a patient expressed: “*If a patient wants to hit someone, at least in VR they hit the air.*” (P4).

### 3.5 Possibilities of VR in treatment according to healthcare providers

To answer the third research question on critical factors to consider when introducing VR in treatment, the opinions and preferences of healthcare providers on how VR could be applied in treatment are provided. The viewpoint of healthcare providers regarding potential applications of VR plays a crucial role in the introduction of VR in practice. By exploring the possibilities and added value of VR, these key areas can be used as a starting point for the introduction of the technology in practice. The codes mentioned by healthcare providers are reported and defined in Table 6.

**Table 6:** Possibilities of VR in treatment according to healthcare providers.

Code	Definition	N*int*^a^	N*tot*^b^
Treatment possibilities with VR			
Observation and assessment	The application of VR to evaluate potential risk factors of patients related to criminal behavior or mental health disorders.	4	4
Practice new behavior and copings skills	The application of VR as a tool for patients to simulate and engage in realistic scenarios where they can practice and refine desired behavior and coping strategies.	3	3
Exposure	The application of VR to simulate and expose individuals to fear- or aggression provoking situations in a controlled and safe environment.	2	2
Integrating VR into daily practice			
Expectation management	The process of informing and setting realistic expectations for patients undergoing VR treatment.	3	3
Existing treatment protocols	Established and standardized procedures, guidelines, or frameworks that healthcare providers follow when providing treatment to patients and that could benefit from VR.	3	3
Part of existing treatment	Integrating VR as a complementary tool within existing treatment frameworks.	1	2
Suitable for all	VR treatment is appropriate and safe for a wide range of patients, without specific exclusion criteria based on their characteristics or conditions.	2	2
Added value	Potential benefits, or positive outcomes that VR can offer patient’s treatment or the therapeutic process, that need to be discussed before the decision on whether a patient can use VR or not.	1	1
Deliberate choice	Conscious and thoughtful decision made about using VR in treatment after careful consideration and discussion within the team of healthcare providers and with the patient.	1	1
Different roles	Distinct responsibilities and contributions that healthcare providers assume within VR treatment.	1	1
Implementation materials and activities			
Materials	Resources, documents, or tools that are developed ant utilized to support the integration of VR into treatment.	6	7
Attention to the possibility of VR	Deliberately considering and exploring the potential applications, benefits and implications of incorporating VR in treatment.	3	3
Intervision groups	Structured and collaborative peer support groups where healthcare providers engage in discussion, reflection, and learning from each other’s experiences in their clinical practice.	2	2
Training sessions	Organized and structured activities designed to provide healthcare providers with knowledge, skills, and competencies necessary to effectively and safely use VR technology in their practice.	1	2
Available time	Allocated timeframe or duration in which healthcare providers need to learn how to use VR and practice with the technology.	1	1
Templates	Pre-designed formats or exercises that serve as a starting point or framework for creating VR scenarios.	1	1
Indication criteria			
Suitable for all	VR treatment is appropriate and safe for a wide range of patients, without specific exclusion criteria based on their characteristics or conditions.	2	2
Added value	Potential benefits, or positive outcomes that VR can offer patient’s treatment or the therapeutic process, that need to be discussed before the decision on whether a patient can use VR or not.	1	1

a The number of interviews the quote was mentioned in (N*int*).

b The number of times the code was mentioned in all interviews with healthcare providers (N*tot*).

#### 3.5.1 Treatment possibilities with VR

Healthcare providers described three ways to use VR in treatment. Firstly, VR can be used as an *observation- and assessment tool*. With VR, insight into patients’ experiences and behavior can be gained. While exposing patients to a virtual scenario, personal triggers or risk factors can be discovered; *“I think you can use it for the initial phase and discover personal triggers.”* (H7)

Secondly, VR can be used to *practice new behaviors and coping strategies*. Healthcare providers mentioned specific applications. For example, when patients are exposed to a challenging situation, they can practice applying stress reduction techniques, and relaxation exercises; *“I am working with a client on stress reduction. We are looking at what causes him stress, for example, big crowds or loud music around him. With all those triggers I can simulate a scenario in which we can practice relaxation exercises.”* (H4). In addition, patients can practice conflict management skills in a role-play that triggers their aggressive behavior. As illustrated by a healthcare provider:

*“You could use it for aggression problems. I think most people will start role-playing. For example, when clients feel threatened on the subway if someone stares at them. With aggression, it is often useful to practice management skills, conflict management.”* (H8)

Finally, VR can be used for *exposure*. Patients can be exposed to situations that trigger their aggressive behavior or personal fear. Within VR, a behavioral experiment can be set up and the healthcare providers and patient can reflect on how the patient acts and feels during the experiment. One healthcare provider illustrated; *“You can use it in the exploratory phase, so to discover signals and triggers. After, you can use it as a replacement for the exposure. You can move much faster in the VR world [than in real-life] to apply exposure and set up more behavioral experiments as a result.”* (H1).

#### 3.5.2 Integrating VR into treatment practice

As can be seen in Table 6, healthcare providers advised on how to embed new VR technology into daily work practice. Several points of attention were mentioned. First, a focus on *expectation management*. According to healthcare providers, patients should be informed about the use of VR early on in their treatment. They need information about the technology itself, the goal, the possibilities, and the working mechanisms. In addition, patients must be informed that they must provide input on personal experiences, triggers, and situations for VR sessions. They should have the opportunity to think along with their healthcare provider and discuss their expectations; *“I think they should slowly get used to VR. I think you should explain very well that you first practice with them and go through it together: What can you expect? That they have a bit of an idea.”* (H5).

Second, healthcare providers mentioned cognitive behavioral therapy and aggression regulation treatment specifically as *existing treatment protocols* to which VR could be a valuable addition. Healthcare providers mentioned that it is important to see VR sessions as an intervention that is used as *part of the existing treatment* plan, not as an independent form of treatment. As one healthcare provider illustrated:

*“It is best to embed it in an existing treatment. I think patients can get used to it slowly. You should explain it very well and go through it together: What it is, what is possible. That they [patients] have an idea and that they can also come up with a situation that you can practice [in VR]”.* (H5)

Healthcare providers addressed indication criteria; patient-related factors that need to be adhered to before patients can use VR in their treatment. Two healthcare providers mentioned that this VR technology could be *suitable for all patients*, since all patients have their own triggers, e.g., a difficult home situation, complex family relationships, or problems in a work environment. Because of the wide variety of possibilities in VR, it applies to most patients’ treatment goals. However, multiple healthcare providers mentioned that VR should not be applied as a standard treatment tool. The *added value* of VR for each and every patient must be discussed with the team of healthcare providers and with the patient. Only if the added value for the patient’s treatment goals is to be expected and it fits with the current treatment plan, VR should be part of their treatment. One healthcare provider illustrated: *“I would not necessarily say in advance that we are going to use VR as standard for everyone. I think that it is very important to look at each and every patient to see whether it fits the treatment goal. Because, for example, with schema therapy treatments you may not need it.”* (H1)

They emphasized that the use of VR should be a *deliberate choice* between the patient and the healthcare provider. The option to use VR should be discussed before treatment starts, at the beginning of the treatment process. The option to use VR and integrate it into a treatment plan should be discussed within a team meeting before being offered as an option to the patient. To illustrate:

*“Pay attention to the option to use VR from the start of the treatment. It is important that it is discussed in a team meeting, while the treatment plan is discussed. I think that it is important that patients are informed about this early on in the treatment. That there is a possibility to use VR. Then you can think about it together with the patient.”* (H2)

Lastly, healthcare providers described that it is important to pay attention to the new skills that are needed to implement VR in the existing workflow. Healthcare providers need to gain skills in *different roles* in order to use VR. They have to create and control the VR session, they have to pay attention to the patient, their emotions, and behavior, all while keeping the treatment goals in mind during the session. As one healthcare provider illustrated, this can increase the threshold for use; *“What I find a barrier in use, is that you have to do a lot. You have to pay attention to the client, control the VR session and you also have to think about what we are going to do.”* (H10).

#### 3.5.3 Implementation materials and activities

A thorough implementation was identified by healthcare providers as an important starting point for working with VR. To help with the integration of VR into existing treatment, healthcare providers expressed the need for several *implementation materials*, activities, or strategies. An introductory video, information brochure, or an online psycho-education module were mentioned by a few healthcare professionals to inform patients and themselves about VR, its possibilities, and its added value. To illustrate; *“Yes, I think it would be a good idea to take a more creative approach, like a video or folder because then they [patients] might also be more stimulated and fascinated to use it. If they have to read a lot, then I think half of them will not do it. Maybe more than half.”* (H4).

In addition to implementation materials, three healthcare providers mentioned that the threshold for use would lower whenever their colleagues and managers would pay more *attention to the possibility of using VR* in treatment. One healthcare provider illustrated; *“The managers have to motivate colleagues and say: Well, this is VR therapy and we think this is an important development. People need to be referred to VR as an option for treatment. It needs more attention so that people think; ‘Oh, there is a VR set? Nice, I’m using it in my treatment plan.”* (H3).

According to the participants, the most important aspect of the implementation of VR is not the materials that are available for patients or healthcare providers; it is the available time they have to practice together with colleagues on how to use VR technology and apply it to treatment. Healthcare providers mentioned that this would increase their self-confidence regarding the use of VR and lower the threshold for actual use:

*“It just takes a lot of time, training, and practice. You will need to practice this in an intervision with colleagues, because it is mainly a lot of ‘doing, doing, doing’, before you have the self-confidence to say: ‘Oh yes, it works, I can do it.’, and actually apply it to treatment with patients. So, I think this is the biggest investment we have to make.”* (H1)

As illustrated above, *intervision groups*, frequent meetings with experienced colleagues, were valued to discuss knowledge on VR, its potential, exchange ideas, address barriers, and share experiences in working with VR technology in treatment with patients. Investing time to practice and organize intervision sessions is seen as important.

In addition to practicing together with colleagues, official *training sessions* on the use of VR and how to set up scenarios were mentioned. In the training, example exercises or *templates* of frequently experienced triggers or situations could be discussed. For example, a role-play script on having an argument with your boss. These prompts could help healthcare providers in setting up a realistic VR scenario. As one healthcare provider mentioned; *“Certain templates that you make available, which are common situations [written down as a VR exercise] which therapists can then perform. That also gives something to hold on to during treatment.”* (H2). To support healthcare providers in the use of VR, VR *experts* or coordinators were mentioned. These experienced colleagues can support healthcare providers who want to apply VR in their treatment by sharing experiences, supervising VR sessions, answering questions, or playing a motivational role.

## 4 Discussion

### 4.1 Principal findings

The study aimed to provide insight into patients’ and healthcare providers’ initial impressions and perspectives regarding the introduction of VR in forensic mental healthcare, to further guide its integration in clinical practice. Patients’ first impressions of VR were predominantly positive, with feelings of enjoyment and curiosity being prominent. They often felt a strong sense of presence, feeling fully immersed in the virtual environment. However, there were also points for improvement noted. The patient’s sense of presence decreased when they focused on unrealistic details in the environment, unnatural movement of the virtual characters, and feelings of discomfort, including dizziness or increased body temperature. Healthcare providers highlighted the importance of personalization in VR scenarios, allowing customization to fit individual patients’ needs and treatment goals. They found the VR software user-friendly and intuitive, emphasizing the ease of use and various possibilities. Some providers expressed a desire for a preview of VR scenarios before immersion and wished for more realistic details in the visual and auditory aspects of the VR environment.

The psychological distress experienced by patients during the VR sessions varied, with most reporting low distress levels at the outset. However, some experienced increased distress during interactions with the virtual environment, particularly when virtual characters engaged with them, leading to feelings of alertness and the need to react. Additionally, the uncertainty of not knowing what to expect during the VR session heightened distress levels, requiring patients to be highly attentive.

For the introduction of VR in practice, patients and healthcare providers highlighted some key points of attention. Emphasis should be placed on the careful introduction of VR to patients, particularly regarding triggering scenarios. Additionally, they stressed the importance of expectation management and shared decision-making between patients and healthcare providers while discussing the option to use VR in treatment. Attention to implementation resources and activities, such as training sessions, intervision groups, and available time to practice with VR were noted for successful implementation. Ultimately, both patients and healthcare providers highlighted the potential benefits and specific applications of VR, such as VR as a tool for exposure to triggering scenarios, practicing new behaviors and coping strategies, or using VR for observation and assessment of risk factors. All while emphasizing its deliberate and well-managed integration into therapeutic practice.

### 4.2 Comparison with prior work

The study’s main findings underscore the positive first impressions of both patients and healthcare providers concerning the potential and application of virtual reality (VR) in treatment. Patients appreciated VR for offering them a valuable opportunity to visually simulate personal experiences, practice new behavior and coping skills, and reflect on this together with their healthcare provider, something not feasible in traditional treatment. This is in line with recent research, where it is previously discussed that the potential of VR offers a unique opportunity to bridge the gap between the safe and controlled treatment environment and the external, often unreachable real world (Botella et al., 2017; Tuente et al., 2020). The potential of VR is evident for both patients and healthcare providers. Consequently, when introducing VR, these stakeholders require neither an extensive persuasion nor an educational campaign to appreciate VR’s utility within the context of treatment. This initial recognition of the technology’s applicability creates a favorable starting point for implementation (Kouijzer et al., 2023).

Beyond these predominantly optimistic evaluations of VR’s benefits and opportunities, patients and healthcare providers indicated a preference for more realistic detail in VR environments and characters. In contrast to the wish for more realistic details for VR to be effective, there are examples of VR applications in healthcare practice that use an abstract environment and are very effective in achieving their goals. As an illustration, Ijsfontein, a software developer specializing in behavior change and learning, designed an application where patients with depression engage in roleplay using highly abstract VR characters. Testing the application with varying degrees of realism revealed that the highly abstract version had the most significant effect on patients. This underscores that an effective application does not need to solely rely on visually realistic detail (Cornet et al., 2019). This is in line with the findings of the current study. Patients expressed a strong sense of presence, the psychological experience of “being there” (Cummings and Bailenson, 2016), even when immersed in VR environments that lacked a satisfactory level of realism according to most participants. Patients felt alert and compelled to respond to the situations in VR. This showed that patients experienced a sense of belonging with a body in the virtual world, emphasizing the process of embodiment and the sense of presence (Ventura et al., 2018).

However, this feeling of alertness also contributed to an increase in patient’s psychological distress during interactions with the virtual environment. For example, most patients experienced more distress when a virtual character imitated speech or motion. This heightened distress was also mentioned to be linked to the uncertainty of the unfamiliar situation patients found themselves in when experiencing VR for the first time. The overarching elevation in psychological distress experienced by all patients can be explained by VR’s capacity to evoke powerful emotional and psychological reactions (González Moraga et al., 2022). Within a therapeutic setting, patients engage with scenarios that provoke their problematic behavior, potentially leading to an even more pronounced impact on psychological distress (González Moraga et al., 2022). Prior investigations have demonstrated that psychological distress and emotional reactions can intensify when personal triggers are incorporated (Botella et al., 2017). This rise in psychological distress and heightened alertness underscores VR’s potential as an instrument for behavior change. However, it also highlights the need for a cautious approach when introducing VR due to its potential intrusiveness, especially when patients are immersed in triggering scenarios. A gradual, incremental approach is advised, commencing with neutral scenarios devoid of visuals or auditory triggers and progressively incorporating triggers and interactions within the virtual scenario.

When introducing VR in treatment, the importance of expectation management should be highlighted. It is essential for healthcare providers to explain and show what patients can expect when they enter a virtual environment. In addition, they could emphasize when introducing VR to patients that while the virtual environment may not look hyper-realistic, it can create a strong sense of presence and generate the associated emotional response. This approach can help manage expectations and reduce potential implementation barriers, which may be largely unfounded, increasing the intention to use VR and successful adoption of the technology (Tamilmani et al., 2021).

Additionally, the decision to incorporate VR into patient treatment should be deliberate and well-considered, involving discussions with both the patient and the healthcare team. Managing expectations is essential, ensuring that patients have a comprehensive understanding of the role VR will play in their treatment and realistic expectations when engaging in VR during treatment sessions. This process of shared-decision making fosters a collaborative environment in which patients and healthcare providers jointly determine the most suitable treatment approach that aligns with patients’ preferences and clinical needs, promoting patient-centered care and enhancing implementation efficiency and treatment adherence (Rogers, 1995; Chong et al., 2013; Kouijzer et al., 2023).

Despite the overarching positive reviews regarding VR, several critical factors require consideration when introducing the technology in practice. It is crucial for healthcare providers and patients to acquaint themselves with the new technology and have access to implementation resources and activities that provide the necessary skills and confidence to employ VR within treatment. This emphasis on skill development and confidence-building aligns with the concept of “trialability” as proposed by Rogers (1995), which underscores the importance of allowing potential adopters to experiment with technology before fully committing to its integration in practice. In this context, facilitating opportunities for healthcare providers and patients to explore VR functionalities, understand its potential benefits, and engage in hands-on experiences can significantly contribute to the successful implementation of VR in healthcare settings.

This study showed the importance of thorough implementation. However, generally, the emphasis on systematic, multi-level implementation is lacking concerning VR’s application in healthcare (Levac et al., 2015; Birckhead et al., 2019; Kouijzer et al., 2023). The current study provides valuable initial insights into important aspects of the introduction of VR technology into treatment practice. It offers points of attention and improvement when introducing a new form of treatment to its end-users.

Yet it also highlights the need for further comprehensive research to fully explore and understand these multifaceted implementation factors. For future directions, placing a heightened emphasis on systematic implementation would be valuable. Adhering to a well-structured implementation framework can empower researchers and practitioners to ensure a comprehensive and well-planned implementation process (Kouijzer et al., 2023). Implementation frameworks such as the Consolidated Framework for Implementation Research (CFIR) (Damschroder et al., 2009) or the Non-Adoption, Abandonment, Scale-Up, Spread, and Sustainability (NASSS) framework (Greenhalgh and Abimbola, 2019) could provide guidance in systematically assessing and addressing implementation challenges and considerations in the context of integrating VR in healthcare. This study serves as a starting point, emphasizing the ongoing journey of exploration required to ensure the effective and sustainable adoption of VR within a forensic healthcare setting.

### 4.3 Strengths and limitations

The strengths of this study lie in its involvement of both patients and healthcare providers, who contributed their initial impressions regarding the introduction of VR into practice. The involvement of end-users and a focus on user experience is especially important when new technology is used (Ventura et al., 2018). Moreover, the deliberate selection of participants from diverse backgrounds and with varying intellectual abilities ensured a comprehensive representation of potential end-users of the VR application. The inclusion of patients with diverse cognitive abilities is especially important, considering that technological interventions must be personalized to the specific needs and capacities of end-users for successful integration into treatment. The study results capture a broad spectrum of perspectives and experiences, providing an overview of essential elements for the introduction of new technology into clinical practice. It facilitates a more informed and efficient integration of VR, enhancing the likelihood of a safe and successful adoption and long-term use of VR, thereby augmenting its value for patients, healthcare providers, and treatment outcomes (van Gemert-Pijnen et al., 2018; Kip et al., 2019a,b).

While it is beneficial for patients to be exposed to VR before the interview, a limitation of this study focuses on the VR immersion in this study not being intended as part of the patient’s treatment. The exposure to VR was primarily meant to gather first impressions as opposed to being an integral part of their treatment program, but it could distort the overall perception of the outcomes as these focus on integrating VR into treatment. In addition, the exposure to VR within this study was comparatively brief, and although patients experienced VR immersion, their exposure was restricted to neutral scenarios devoid of personal triggers. This aspect might have limited the complete potential impact of VR on participants. Therefore, it is important to consider VR’s long-term effects on treatment and the exploration of its potential beyond the scenarios investigated in this study. Undertaking a more comprehensive investigation, involving longitudinal evaluations of outcome measures, could furnish a more complete understanding of the enduring benefits and potential limitations of VR within the context of forensic psychiatry.

An additional limitation to consider is that patients and healthcare providers involved in this study all volunteered to participate. A selection bias might have taken place, resulting in participants that generally held a favorable attitude toward the integration of VR in treatment. This could restrict the generalizability of the findings. The study sample might not be representative of the broader population, and therefore, the results may not capture the full range of perspectives and experiences related to VR use in forensic healthcare. Therefore, the results should be interpreted with caution and placed in a broader perspective. It might prove beneficial to track a diverse group of healthcare providers and their patients as they engage with VR in practice when the technology is successfully implemented. This approach would yield a broader understanding of implementation barriers and points of attention in the use of VR.

In addition to the potential of VR in forensic mental healthcare, as is discussed in this study, the limitations of applying the technology in mental healthcare should also be considered as well. Ethical considerations, for example, particularly in discussions surrounding the exposure of vulnerable populations should be taken into account. VR can be used to provoke emotions of anger and aggression. When eliciting physical and verbal anger, it can be questioned to what extent the patient may be stimulated in eliciting these emotions. The strength of VR is that reality can be realistically simulated, but the possibly provoked intense emotions and aggressive behavior need to be taken into account when integrating technology into mental healthcare (Kip et al., 2024). Finally, as shown above, the generalizability of findings from VR implementation studies poses a challenge, emphasizing the importance of diverse sample populations in research to draw broader conclusions about the efficacy and impact of VR implementation interventions in mental healthcare. These considerations highlight the nuanced approach required when integrating VR technology into clinical practice, needing careful navigation of ethical, technical, clinical, and research-related challenges.

## 5 Conclusion

The integration of VR into forensic mental healthcare holds great potential for behavior change. However, its immersive characteristics also increase the chance of amplifying psychological distress. This emphasizes the need for caution when using VR– especially when a vulnerable patient group is subjected to triggering scenarios. This study advocates for a gradual introduction of the technology and provides valuable insights into key elements for this introduction in clinical practice. Personalized treatment plans, developed collaboratively between patients and healthcare providers, and shared decision-making in setting up and integrating VR in treatment are crucial for navigating the introduction of VR effectively. Healthcare providers require adequate training and support to confidently use VR, in which attention should be paid to managing expectations for patients and providing them with adequate support throughout the introduction and integration of VR in treatment. While these key elements are well-known and should be considered as standard practice, they are not always applied when integrating a new form of technology in treatment. It is important to take these elements into account when introducing technology, particularly when the technology is used as a powerful tool to change behavior in vulnerable patient populations. This study highlights that even the initial step of integrating VR into practice – the introduction phase – demands careful planning and a personalized approach. This underscores the need for ongoing refinement and a systematic approach to the overall implementation of VR. These efforts are crucial to fully realize its potential in clinical practice.

## Data availability statement

The raw data supporting the conclusions of this article will be made available by the authors, without undue reservation.

## Ethics statement

The studies involving humans were approved by Ethics Committee of the University of Twente (Behavioral, Management, and Social Sciences, number 210645). The studies were conducted in accordance with the local legislation and institutional requirements. The participants provided their written informed consent to participate in this study.

## Author contributions

MK: Writing – original draft. HK: Writing – review & editing, Supervision. SK: Writing – review & editing. YB: Writing – review & editing.

## Appendix

Tables A1, A2.

**Table A1:** Semi-structured interview scheme for interviews with patients.

Questions for reflection during the immersion in every scenario	
What did you think of it?	RQ1
What did it feel like?	RQ1 + 2
On a scale of 1 to 10, with 1 being completely relaxed and 10 experiencing extreme stress, how high was your level of distress?	RQ2
Questions for evaluation post-immersion	
What is your first impression of VR?	RQ1
What did you like or positively appreciate about VR?	RQ1
Are there any things you did not like or would improve in VR?	RQ1
How realistic/authentic did it feel?	RQ1
What did you notice about yourself in VR? When did you notice this?	RQ1 + 2
What was your level of distress during the VR scenarios?	RQ2
What stood out to you while you were in VR?	RQ1
Would you be willing to use VR in treatment? Why (not)?	RQ3
What should we consider when introducing VR to patients?	RQ3

**Table A2:** Semi-structured interview scheme for interviews with healthcare providers.

What is your initial impression of using the VR dashboard?	RQ1
What did you find challenging about using the VR dashboard? How would you evaluate the usability of the VR dashboard? How would you evaluate the design of the VR dashboard? To what extent do you understand the use of the VR dashboard?	RQ1
Do you need more information on the dashboard to build a VR scenario? If so, what information do you think is currently missing?	RQ1
What would you like to change or add to this dashboard to improve it?	RQ1
Are there any (content-related) environments, characters or triggers that are missing so far?	RQ1
To what extent would you like to use this VR application in treatment? How would you apply this VR application in treatment? Within which existing treatment protocols would you apply VR? What would that look like in practice? Can you give examples?	RQ3
What are additional points of attention in terms of timing, introduction, and explanation that are needed to introduce VR to patients?	RQ3
Would you prefer any implementation activities or materials, such as training or protocols to support you during the introduction of VR in treatment? What kind of implementation activities/materials would you prefer? What would that look like in practice? Can you give examples?	RQ3
