# Supplementary material for: The introduction of virtual reality in forensic mental healthcare – an interview study on the first impressions of patients and healthcare providers regarding VR in treatment
Source: Front Psychol. 2024 Apr 30;15:1284983. doi: 10.3389/fpsyg.2024.1284983 (PMC11091295; doi:10.3389/fpsyg.2024.1284983)
Supplement: Supplementary file 1 [file Data_Sheet_1.PDF]

COREQ (COnsolidated criteria for REporting Qualitative research) Checklist

A checklist of items that should be included in reports of qualitative research. You must report the page number in your manuscript where you consider each of the items listed in this checklist. If you have not included this information, either revise your manuscript accordingly before submitting or note N/A.

Topic	Item No.	Guide Questions/Description	Response	Reported on Page No.
**Domain 1: Research team and reflexivity**				
Personal characteristics				
Interviewer/facilitator	1	Which author/s conducted the interview or focus group?	1st Author (MK)	24
Credentials	2	What were the researcher's credentials? E.g. PhD, MD	Master of Science (Health Psychology), PhD.	-
Occupation	3	What was their occupation at the time of the study?	Researcher	-
Gender	4	Was the researcher male or female?	Female	-
Experience and training	5	What experience or training did the researcher have?	Experience of seven years as a researcher	
Relationship with participants				
Relationship established	6	Was a relationship established prior to study commencement?	Yes	-
Participant knowledge of the interviewer	7	What did the participants know about the researcher? e.g. personal goals, reasons for doing the research	Her occupation and reasons for carrying out the research.	-
Interviewer characteristics	8	What characteristics were reported about the inter viewer/facilitator? e.g. Bias, assumptions, reasons and interests in the research topic	Occupation, reasons and interests in this research.	7
**Domain 2: Study design**				
Theoretical framework				
Methodological orientation and Theory	9	What methodological orientation was stated to underpin the study? e.g. grounded theory, discourse analysis, ethnography, phenomenology, content analysis	Content analysis	8
Participant selection				
Sampling	10	How were participants selected? e.g. purposive, convenience, consecutive, snowball	Purposive and convenience sampling	6
Method of approach	11	How were participants approached? e.g. face-to-face, telephone, mail, email	Face-to-face (patients) and via email (healthcare providers)	6
Sample size	12	How many participants were in the study?	18 (10 healthcare providers, 8 patients)	6+7

Non-participation	13	How many people refused to participate or dropped out? Reasons?	None	6
Setting				
Setting of data collection	14	Where was the data collected? e.g. home, clinic, workplace	Clinic (patients) and via Zoom (healthcare providers)	8
Presence of non-participants	15	Was anyone else present besides the participants and researchers?	A healthcare provider during the patient interviews	8
Description of sample	16	What are the important characteristics of the sample? e.g. demographic data, date	See table on page 7.	7
Data collection				
Interview guide	17	Were questions, prompts, guides provided by the authors? Was it pilot tested?	Questions and prompts were provided. The interviews were pilot tested.	7+8
Repeat interviews	18	Were repeat inter views carried out? If yes, how many?	No	-
Audio/visual recording	19	Did the research use audio or visual recording to collect the data?	Audio recording (healthcare providers) and visual recording (of VR session with patients)	8
Field notes	20	Were field notes made during and/or after the inter view or focus group?	No	-
Duration	21	What was the duration of the inter views or focus group?	Patient interviews: average of 23 minutes. Healthcare provider interviews: average of 47 minutes	8
Data saturation	22	Was data saturation discussed?	Yes	8
Transcripts returned	23	Were transcripts returned to participants for comment and/or	No	-
**Domain 3: analysis and findings**				
Data analysis				
Number of data coders	24	How many data coders coded the data?	Author 1 and checked by Author 2	8
Description of the coding tree	25	Did authors provide a description of the coding tree?	Yes (See tables in result section)	8-18
Derivation of themes	26	Were themes identified in advance or derived from the data?	Derived from data	8
Software	27	What software, if applicable, was used to manage the data?	-	-
Participant checking	28	Did participants provide feedback on the findings?	No	-
Reporting				
Quotations presented	29	Were participant quotations presented to illustrate the themes/findings? Was	Yes (see result section)	9-18

		each quotation identified? e.g. participant number		
Data and findings consistent	30	Was there consistency between the data presented and the findings?	Yes	9-18
Clarity of major themes	31	Were major themes clearly presented in the findings?	Yes	9-18
Clarity of minor themes	32	Is there a description of diverse cases or discussion of minor themes?	Yes	9-18

Developed from: Tong A, Sainsbury P, Craig J. Consolidated criteria for reporting qualitative research (COREQ): a 32-item checklist for interviews and focus groups. *International Journal for Quality in Health Care*. 2007. Volume 19, Number 6: pp. 349 – 357

Once you have completed this checklist, please save a copy and upload it as part of your submission. DO NOT include this checklist as part of the main manuscript document. It must be uploaded as a separate file.
